# Quantification of Isomaltulose in Food Products by Using Heteronuclear Single Quantum Coherence NMR-Experiments

**DOI:** 10.3389/fnut.2022.928102

**Published:** 2022-06-27

**Authors:** Lea Fels, Franziska Ruf, Mirko Bunzel

**Affiliations:** Department of Food Chemistry and Phytochemistry, Institute of Applied Biosciences, Karlsruhe Institute of Technology (KIT), Karlsruhe, Germany

**Keywords:** NMR, HSQC, ASAP-HSQC, non-uniform sampling, carbohydrates, isomaltulose, quantification

## Abstract

Isomaltulose is a commonly used sweetener in sports nutrition and in products intended for consumption by diabetics. Because previously established chromatographic methods for quantification of isomaltulose suffer from long analysis times (60–210 min), faster quantitative approaches are required. Here, an HSQC (heteronuclear single quantum coherence) experiment with reduced interscan delay was established in order to quantify isomaltulose next to potential additional sugars such as d-glucose, d-fructose, d-galactose, sucrose, lactose, and maltose in 53 min. By using HSQC coupled to non-uniform sampling (NUS) as well as ASAP-HSQC (acceleration by sharing adjacent polarization), analysis times were reduced to a few minutes. Application of NUS-HSQC with reduced interscan delay takes 27 min, resulting in accurate and precise data. In principle, application of ASAP-HSQC approaches (with analysis times as low as 6 min) can be used; however, precision data may not suffice all applications.

## Introduction

Many foods contain high levels of sugars as sweeteners. A high consumption of sugars is potentially linked to the occurrence of type-2 diabetes (1); however, this discussion is still open and different sugars differently affect blood glucose levels. Increased consumption of, for example, glucose and, to a lower degree, sucrose causes the blood glucose level to rise rapidly. In patients who suffer from diabetes it cannot be leveled out quickly due to low amounts of produced insulin or cells' insulin resistance (2, 3). Thus, frequent consumption of high or even moderate glycemic foods by diabetic people results in chronic hyperglycemia, which can lead to many serious symptoms such as visual disturbances, stroke, kidney damage, and even coma (3). Therefore, it is important for diabetic patients to consume sugars that have a lower impact on blood glucose levels than sucrose or glucose. Also, preventing the progression of prediabetes to type 2 diabetes requires - besides general lifestyle adaptations - changes in the diet. Thus, usage of isomaltulose has become more popular in many products. Plasma insulin concentrations are 30–50 % lower after consumption of isomaltulose, and glucose is provided up to 1 h longer compared to consumption of an equivalent amount of sucrose (4). These two effects help in particular diabetic patients to keep their blood glucose levels at constant levels for longer periods of time. However, healthy people may also benefit from consuming isomaltulose. When isomaltulose was consumed immediately before or during physical exercise, both increased performance and better recovery were observed (4). The taste and texture of sucrose and isomaltulose are very similar, with isomaltulose having only half the sweetening power of sucrose. However, isomaltulose is much more stable than sucrose under acidic conditions (5, 6). Because of these favorable properties, isomaltulose is an often used ingredient in sports foods and sports drinks. Also, many weight loss products are sweetened with isomaltulose.

Due to the increasing usage of isomaltulose in food products, accurate, precise, and rapid methods are needed to quantify isomaltulose, thus verifying declaration of the products. Up to now, liquid chromatographic methods have been preferred for the quantitative analysis of isomaltulose. In Germany, application of high-performance liquid chromatography with refractive index detection (HPLC-RI) has been established as the official method to quantify isomaltulose (7). In addition, high-performance anion exchange chromatography with pulsed amperometric detection (HPAEC-PAD) has been recommended by the Association of Analytical Chemists (AOAC) for the quantification of food-relevant carbohydrates such as isomaltulose (8). Also, sugars in foods can be determined by using gas chromatographic methods with flame ionization detection (GC-FID), although this approach requires additional derivatization. The analytes are oximated and subsequently derivatized to trimethylsilyl ethers. Oximation results in two signals for reducing sugars, their syn- and anti-isomers (9, 10). However, derivatization is time-consuming and involves the use of reagents that are toxic to humans and/or the environment. None of the currently established methods can be applied to determine isomaltulose in food in short analysis times.

One-dimensional NMR experiments are characterized by their fast analysis times. They have been used for the quantification of nutritionally relevant sugars, although their resolution is usually not sufficient for direct quantification and they are therefore more likely to be coupled with multivariate assessment strategies (11). Increased resolution can be achieved by using two-dimensional NMR experiments such as the heteronuclear single quantum coherence (HSQC) experiment. This experiment is particularly suitable for the analysis of sugars because CH coupling signals are detected, and carbons in sugars are protonated (12). However, HSQC experiments require up to several hours to record, resulting in no gain in analysis time over previously established comparative methods.

The interscan delay has a large impact on the quantification of analytes by NMR experiments, but also on the duration of the experiments. It should be sufficiently long to allow all nuclei to return to the initial state before the next scan and therefore to achieve maximum signals (13, 14). In practice, interscan delays of three to five times the length of the longitudinal relaxation time of the slowest relaxing nucleus have become established for quantification. However, reducing the interscan delay can also shorten the measurement time. We demonstrated in a previous work that HSQC experiments with lower interscan delays can be used to quantify d-glucose, d-fructose, d-galactose, sucrose, lactose, and maltose in dairy products, thereby significantly reducing experiment times (15). Non-uniform sampling (NUS) was also applied to shorten the measurement time by recording only a randomly distributed portion of data points (15). The missing data points are added after the measurement by reconstruction algorithms (16). An additional reduction of the measurement time can be achieved by using the measurement time reduced acceleration by sharing adjacent polarization (ASAP)-HSQC method, which generates HSQC spectra that are sufficiently resolved for quantification in a few minutes (15, 17).

In this work, HSQC methods were applied to quantify isomaltulose as well as simultaneously other monosaccharides and disaccharides. Measures such as shortening the interscan delay, use of NUS, as well as application of the ASAP-HSQC pulse sequence were tested to reduce analysis time. The analytes were quantified in sports foods as well as in weight loss and weight maintenance products.

## Materials and Sample Preparation

### Chemicals and Enzymes

All chemicals and non-deuterated solvents were either from Sigma-Aldrich (St. Louis, USA), Carl Roth (Karlsruhe, Germany), or VWR (Radnor, USA). Phenyl-β-d-glucopyranoside (98 %) was from Alfa Aesar (Kandel, Germany). Deuterium oxide (99.9 %) was obtained from Deutero (Kastellaun, Germany), and enzymatic assay kits were purchased from Roche Diagnostics (Basel, Switzerland).

### Reference Materials and Food Samples

d-Glucose (≥99.5 %), d-galactose (≥99 %), d-fructose (≥99 %), l-arabinose (99 %), lactose monohydrate (≥99.5 %), and maltose monohydrate (≥99 %) were purchased from Sigma Aldrich (St. Louis, USA), sucrose (≥99.5 %) from Carl Roth (Karlsruhe, Germany), isomaltulose hydrate (98 %) from Acros Organics (Geel, Belgium), and maltulose monohydrate (≥98.0 %) from Carbosynth (Compton, UK). The following food products were purchased on the internet trade: product **1** – a food supplement made of pure isomaltulose; product **2** - a food supplement containing isomaltulose as well as various amino acids, and plant extracts; product **3** – a carbohydrate gel used to provide energy during long training sessions, product **4/5** – a powder to be mixed with water to form a shake, used as a meal replacement for weight loss, chocolate (product **4**) and strawberry (product **5**) flavors; product **6** – a milk-based shake used as a meal replacement; product **7** – a bar made of edible insects, sesame seeds, and almonds, marketed as a snack.

### Sample Preparation

To perform **enzymatic analyses**, the **HPAEC-PAD** method, and the **GC-FID** method, the samples were processed as follows: samples **1**, **2**, and **3** were only dissolved and diluted with double distilled water to reach the working range of the respective method. Products **4–7** were clarified by successive addition of A) aqueous potassium hexacyanoferrate (II) solution (150 g/L) and B) aqueous zinc sulfate solution (300 g/L). In detail, product **6** (12.0 g) was mixed with 2 mL each of A) and B), products **4** and **5** (4.0 g each) with 5 mL each of A) and B), and product **7** (1.0 g, ground) with 1 mL each of A) and B). The pH was adjusted to 7.5–8.0 with sodium hydroxide solution. Following centrifugation (9,392 g, 5 min), the supernatant was removed, the residue was washed with water, and the supernatants were combined. The volume was made up to 50 mL in a volumetric flask. Before measurement, an aliquot was filtered through a syringe filter (Teflon, 0.45 μm) and diluted according to the working range of the method used.

For **HSQC-NMR** based analyses, samples **1–3** were simply dissolved in potassium phosphate buffer. To prepare the 0.1 M potassium phosphate buffer, an aliquot of a potassium dihydrogen phosphate solution (13.6 g/L) was mixed with 1.5 times the volume of a dipotassium hydrogen phosphate solution (17.4 g/L) and adjusted to pH = 7.0. Sample **1** (0.25 g), sample **2** (1.25 g), and sample **3** (10 g) were added to a flask, and the volume was made up to 25 mL with the buffer solution. An aliquot was filtered through a syringe filter (Teflon, 0.45 μm), and 0.1 mL of a solution of maltulose in potassium phosphate buffer (80 mg/mL) was added to 0.9 mL of the filtered aliquot. Before NMR measurements, 10 % deuterium oxide (for the lock signal) and 0.5 μL acetone [for spectral calibration (^1^H δ = 2.22 ppm, ^13^C δ = 30.89 ppm) (18)] were added. Products **4–7** were clarified following the Carrez procedure. In detail, products **4** and **5** (4.0 g each) were mixed with 5 mL of A) and B) (for further details on Carrez solutions see above), product **6** (4.5 g) with 0.75 mL of each Carrez solution, and product **7** (1.0 g) with 1 mL each of A) and B). The pH was adjusted to 7.0 with sodium hydroxide solution. After centrifugation, the Carrez procedure described above was further applied. To 0.9 mL of the filtered aliquots of products **4 – 7** 0.1 mL of aqueous maltulose solution (80 mg/mL) was added. Before NMR measurements, 10 % deuterium oxide and 0.5 μL acetone were added.

## Methods

### Reference Methods

#### Enzymatic Assays

The following enzymatic assay kits were used and carried out according to the manufacturer's specifications: d-glucose/ d-fructose and lactose/ d-galactose.

#### HPAEC-PAD

d-Glucose, d-galactose, d-fructose, and sucrose were analyzed according to Fels and Bunzel (15). Isomaltulose was analyzed by applying a method that was previously published to quantify lactose and maltose (15). Calibration was carried out using mixtures of the analytes d-glucose, d-galactose, d-fructose, sucrose, lactose, maltose, and isomaltulose in the concentration range from 1.0 mg/L to 15.0 mg/L (six calibration points), each containing l-arabinose as internal reference (7.5 mg/L). Analytes were quantified by determining integral ratios (analyte/internal reference). The calibration curve followed a quadratic regression.

#### GC-FID

The method is based on a two-step derivatization of the saccharides in the solvent pyridine to obtain more volatile trimethylsilyloximes (TMSO). Oximation of the saccharides was achieved by treatment of the sample solution with a 2.5 % hydroxylammonium chloride solution at room temperature, resulting in fixation of the reducing saccharides in their open-chain form (syn- and anti-isomers). In the second step, silylation was carried out by using *N*-methyl-*N*-trimethylsilyltrifluoroacetamide (MSTFA) and chlorotrimethylsilane under heat. Phenyl-β-d-glucopyranoside was used as internal standard. For reducing saccharides, the sum of the two individually evaluated peak areas of the TMSO isomers was used for calculations, if possible. Analytes were quantified by determining integral ratios (analyte/internal reference). The calibration curve followed a linear regression.

### NMR Experiments

NMR spectroscopy was carried out on an Ascend 500 MHz spectrometer (Bruker Biospin, Ettlingen, Germany) equipped with a Prodigy cryoprobe. Calibration was carried out using mixtures of the analytes d-glucose, d-galactose, d-fructose, sucrose, lactose, maltose, and isomaltulose in the concentration range from either 1.0 g/L to 15.0 g/L containing 8 g/L maltulose as internal reference or 0.8 g/l to 12.1 g/L containing 6 g/L maltulose. Six calibration points were used. Analytes were quantified by determining integral ratios (analyte/internal reference). The calibration curve followed a linear regression. All spectra were recorded at 25°C. The standard Bruker HSQC pulse sequence “hsqcetgp” was used. The ASAP-HSQC pulse program (asap_hsqc_sp_bruker) was provided by the working group of Prof. B. Luy (Karlsruhe Institute of Technology (KIT), Karlsruhe, Germany). Two scans were monitored; the spectral width was 4.50 ppm acquiring 1,024 data points (acquisition time (AQ) = 0.227 s) in the ^1^H dimension and 100.00 ppm using 1,024 data points (AQ = 0.041 s) in the ^13^C dimension. Interscan delays (D_1_) of 1.5 s and 6.0 s/7.0 s were tested in the HSQC experiment. In the ASAP-HSQC experiment D_1_ could be reduced to 0.05 s. When using NUS, a 50 % NUS level, unweighted sampling, and the reconstruction algorithm IST were applied. Linear prediction and zero filling (2,048 datapoints) were performed in both dimensions as well as a cosine bell apodization as a weighting function. Manual phase correction and automatic baseline correction were applied. After NUS reconstruction, a Hilbert transformation in the indirect dimension was used to allow phase correction (15).

Longitudinal relaxation times were acquired with aqueous solutions of the individual analytes (c = 5.0 g/L), mixed with 10 % deuterium oxide. The standard Bruker pulse program “t1ir” (inversion recovery experiment) was used with 16 scans, the spectral width was 19.99 ppm, and AQ was 0.819 s.

**Figure 1:**
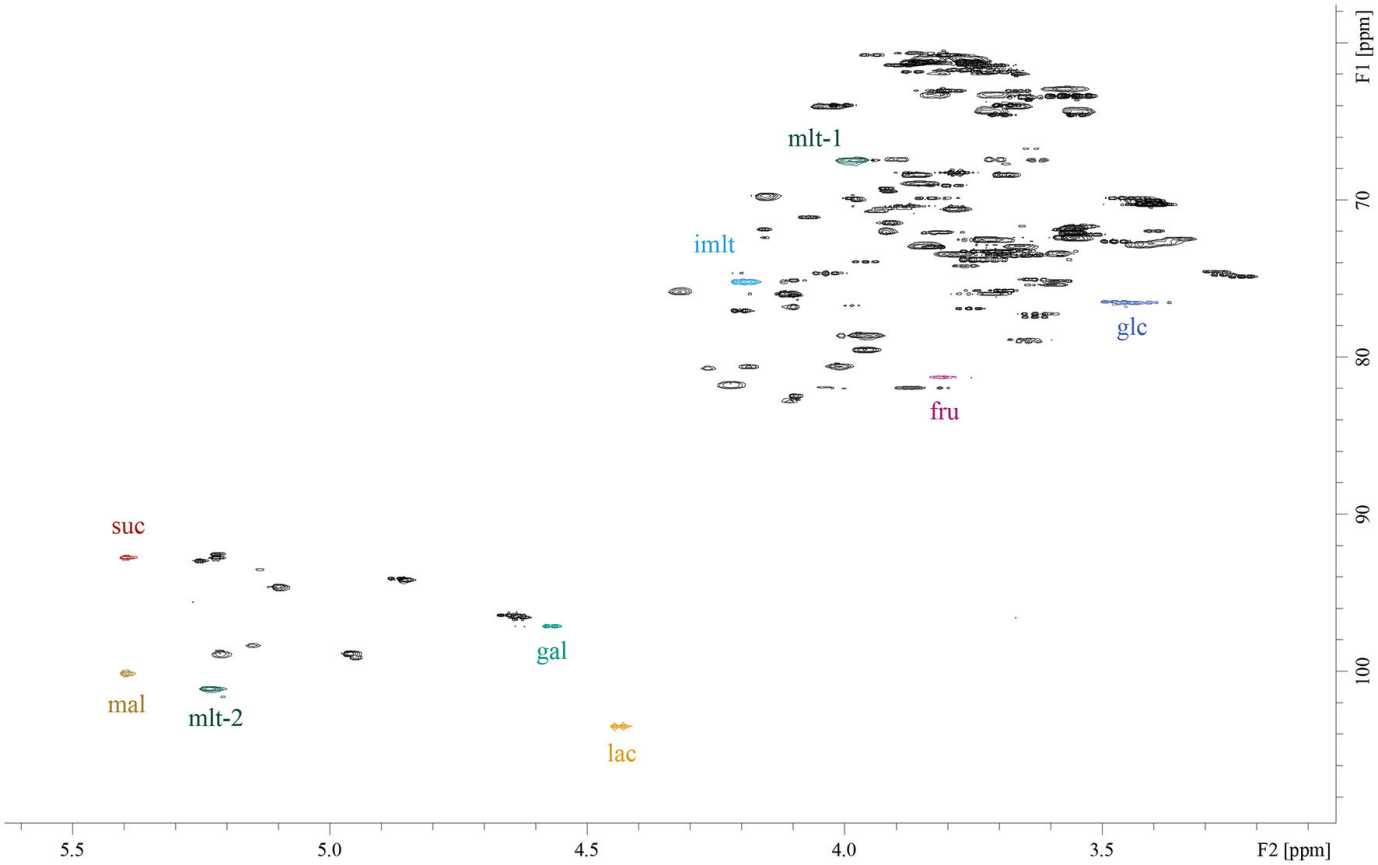
HSQC-spectrum of an aqueous solution of the sugars d-glucose (glc), d-galactose (gal), d-fructose (fru), sucrose (suc), lactose (lac), maltose (mal), isomaltulose (imlt), and maltulose (mlt). Chemical shifts of the selected signals for quantification are quoted in the Section NMR Experiments. The measurement was carried out with the following acquisition parameters: pulse sequence “hsqcetgp”, two scans, size of time domain 1,024 in both dimensions, D_1_ = 1.5 s. HSQC: heteronuclear single quantum coherence.

The following ^1^H/^13^C correlation signals were selected for quantification (see also Figure 1): d-glucose 3.47 / 76.47 ppm; d-galactose 4.58/ 97.14 ppm; d-fructose 3.81/ 81.26 ppm; sucrose 5.40/ 92.78 ppm; lactose 4.45/ 103.62 ppm; maltose 5.39/ 100.14 ppm, isomaltulose 4.20/ 75.17 ppm, maltulose-1 3.98/ 67.46 ppm and maltulose-2 5.23/ 101.11. The signal maltulose-1 was used for HSQC experiments and maltulose-2 for ASAP-HSQC experiments due to low signal-to noise ratios of maltulose-1 (S/N <9). The signals were selected because they do not overlap with any other food-related sugar signals or other signals from the matrices and are sufficiently resolved (Figure 1). 2D-volume integrals were determined by manual integration using TopSpin version 4.0.2 (Bruker Biospin, Ettlingen, Germany).

### Recovery Experiments

To perform recovery experiments, half the sample volume of product **3** was spiked with isomaltulose, so that the final concentration of isomaltulose matched the content in the total sample volume. Subsequently, the samples were prepared according to Section Sample Preparation and diluted as required by the used method.

### Statistics

Statistical evaluation of data of product **3** that were generated by the different methods (see Figure 4, Supplementary Table S3) was performed by applying a one-factor analysis of variance in combination with a *post-hoc* Tukey test in Origin 2019. All other products were analyzed either in triplicate (HSQC experiments) indicating the standard deviation or in duplicate (reference methods) using the half range to demonstrate the spread of the data.

## Results and Discussion

Because isomaltulose is not necessarily used as the sole sugar in food products, the quantification of isomaltulose next to sugars such as d-glucose, d-fructose, d-galactose, sucrose, lactose, and maltose was deemed necessary. Previously optimized HSQC acquisition and processing parameters were used for initial measurements of isomaltulose, individually and in combination with additional sugars (15). Isomaltulose shows signals in the HSQC spectrum that are nicely separated from diagnostic signals of other sugars. As shown in Figure 1, the correlation signal ^1^H4/^13^C4 of the β-fructose unit at 4.20/ 75.17 ppm was chosen for quantitative purposes. Due to its structural similarity to isomaltulose, maltulose was used as an internal standard, and the signals ^1^H3/^13^C3 of the β-fructose unit (pyranose) at 3.98/ 67.46 ppm and ^1^H1/^13^C1 of the glucose unit at 5.23/101.11 ppm were picked for quantification by using “regular” or ASAP-HSQC experiments, respectively.

**Figure 2:**
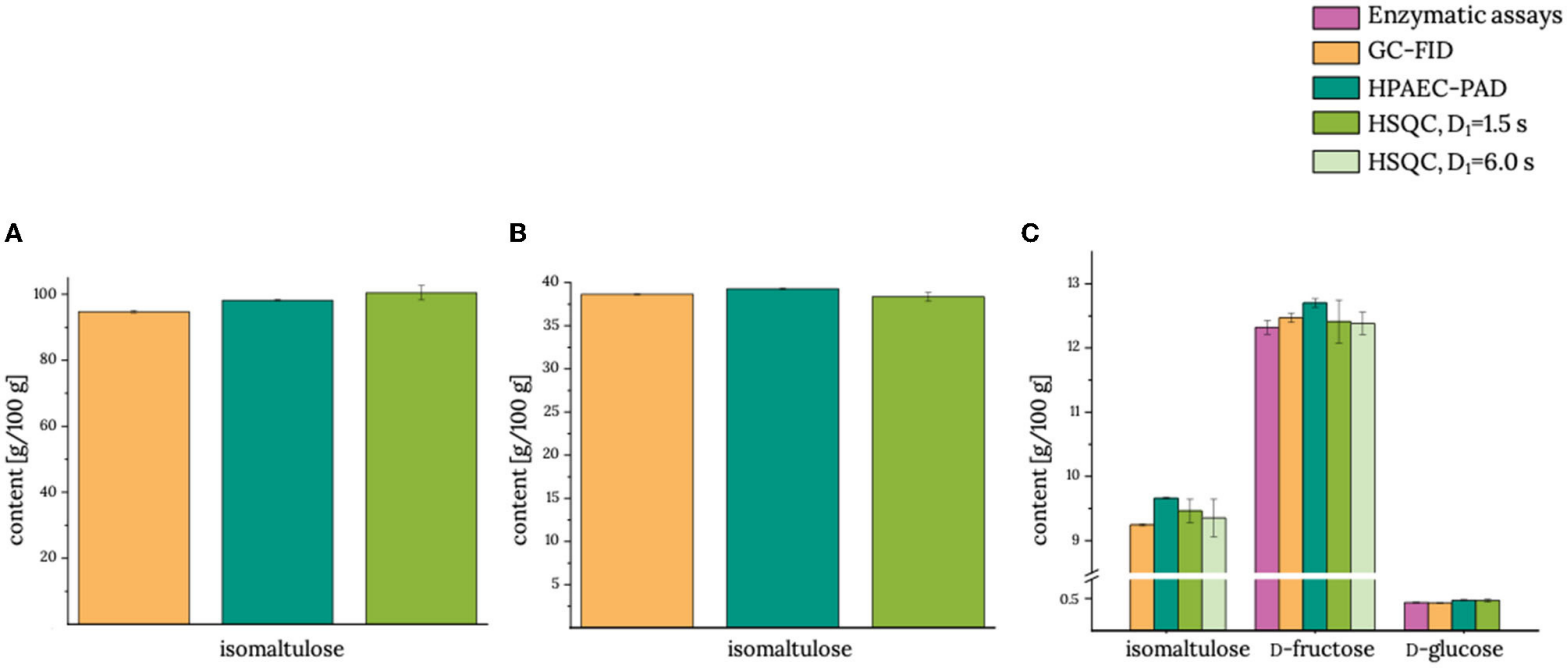
Contents of isomaltulose and other sugars (if applicable) in **(A)** product **1**, **(B)** product **2**, and **(C)** product **3**. GC-FID, gas chromatography with flame ionization detector; HPAEC-PAD, high-performance anion-exchange chromatography with pulsed amperometric detection; HSQC, heteronuclear single quantum coherence; D_1_, interscan delay. Analyses by using the reference methods were performed in duplicate using the half range to demonstrate the spread of the data. HSQC measurements were performed in triplicate determination, and the standard deviation is given. Actual data are given in Supplementary Table S2.

Currently established methods to analyze sugars in general such as enzymatic, HPAEC-PAD, and GC-FID approaches require analysis times of more than 60 min per sample. To be competitive with these methods or, ideally, to be faster than these methods, the HSQC experiments have to be optimized. Otherwise, a 2D-NMR approach would not be competitive simply due to the inherently low sensitivity of NMR spectroscopy, which can, theoretically, be improved by using more scans, higher fields, or cooled probes (19). However, sensitivity (signal-to-noise) is only increased with the square root of the number of scans. Incorporation of NUS into HSQC experiments and optimization of interscan delays are suitable tools to significantly reduce the measurement time of correlation experiments. The developed HSQC methods with shortened interscan delays, NUS coupling as well as the established ASAP-HSQC methods can achieve a significant reduction of the measurement time (Table 1) as detailed in the following: First, isomaltulose and other sugars (if applicable) of the food products were quantified, simply dissolved in buffer solution (Figure 2). Because there is no enzymatic approach for the determination of isomaltulose, only d-fructose and d-glucose were determined enzymatically in product **3**. In addition to the HSQC method that uses a reduced interscan delay of D_1_ = 1.5 s, an interscan delay of three times the length of the slowest relaxing nucleus was used to analyze product **3** for longitudinal relaxation times (see Supplementary Table S1).

**Table 1:** Experiment times (t_Exp_) for the quantification of d-glucose, d-galactose, D-fructose, sucrose, lactose, maltose, and isomaltulose with the described methods.

**Method description**	**t_exp_**
Enzymatic assays (several assays necessary, quantification of isomaltulose not possible)	~60 min per assay
HPAEC-PAD (separation on two different columns necessary)	73 min and 60 min
GC-FID	
Derivatization	180 min
GC-run	30 min
HSQC, D_1_ = 1.5 s	53 min
HSQC, D_1_ = 1.5 s, 50 % NUS	27 min
HSQC, D_1_ = 6.0 s / D_1_ = 7.0 s	208 min / 242 min
HSQC, D_1_ = 6.0 s, 50 % NUS	105 min
ASAP-HSQC	12 min
ASAP-HSQC, 50 % NUS	6 min

*HPAEC-PAD, high-performance anion-exchange chromatography with pulsed amperometric detection; GC-FID, gas chromatography with flame ionization detection; HSQC, heteronuclear single quantum coherence; D_1_, interscan delay; NUS, non-uniform sampling; ASAP, acceleration by sharing adjacent polarization*.

In all three products that were tested without additional clarification as a clean-up procedure (products **1–3**), the isomaltulose content determined with the HSQC method with D_1_ = 1.5 s was equivalent to those that were analyzed by using our reference methods. Moreover, the determined isomaltulose content did not change when the larger interscan delay of 6.0 s was applied (product **3**). The same held true for d-fructose, which was exemplarily re-analyzed here (15). Thus, shortening of the interscan delay to 1.5 s in the HSQC experiment did not negatively affect the quantification of isomaltulose.

**Figure 3:**
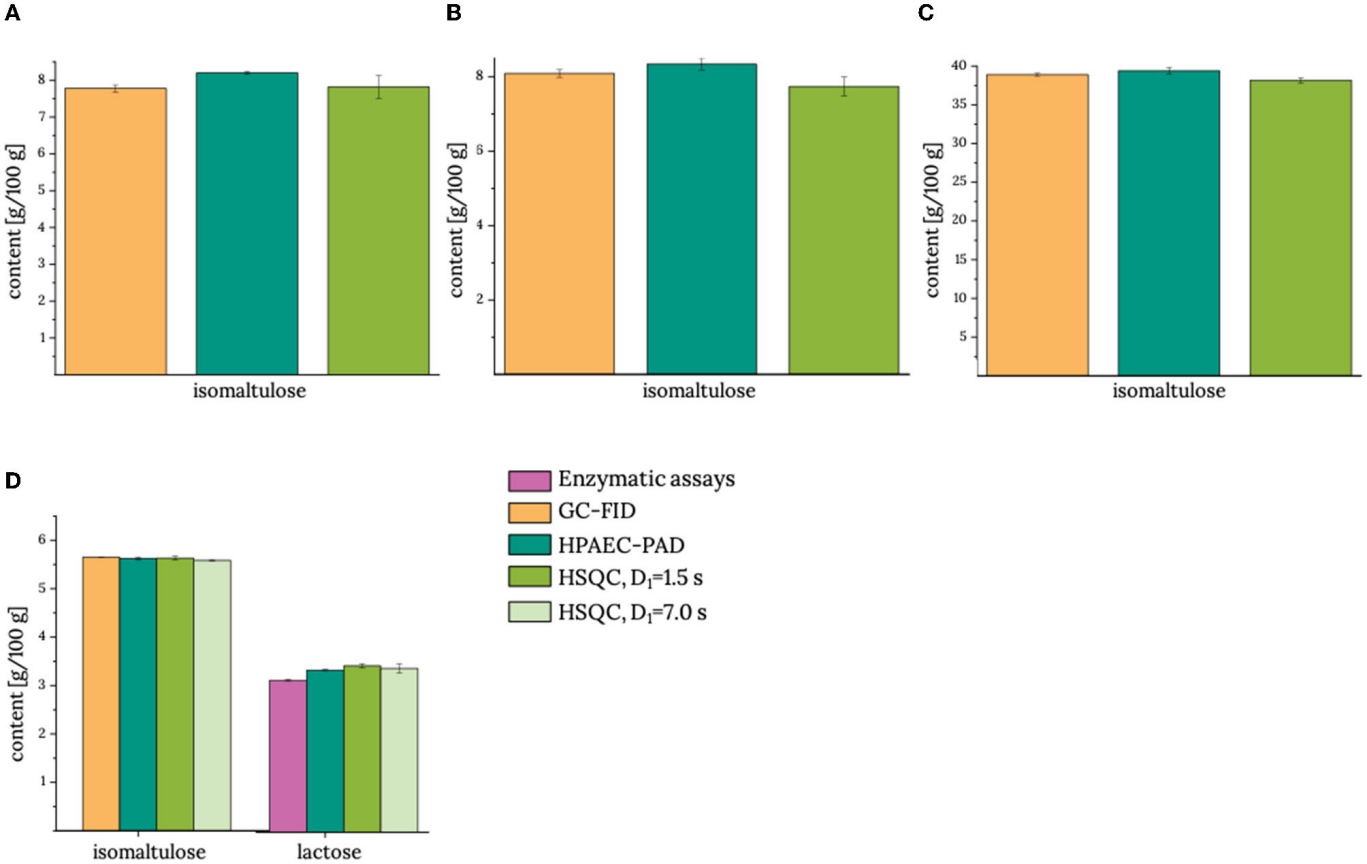
Contents of isomaltulose and (if applicable) lactose in **(A)** product **4**, **(B)** product **5**, **(C)** product **7**, and **(D)** product **6**. GC-FID: gas chromatography with flame ionization detector; HPAEC-PAD: high-performance anion-exchange chromatography with pulsed amperometric detection; HSQC, heteronuclear single quantum coherence; D_1_, interscan delay. Analyses by using the reference methods were performed in duplicate using the half range to demonstrate the spread of the data. HSQC measurements were performed in triplicate determination, and the standard deviation is given. Actual data are given in Supplementary Table S2.

In the next step, isomaltulose was analyzed in more complex food products that required Carrez-clarification as a clean-up step (Figure 3). As product **6** contained lactose as additional sugar, an enzymatic assay was used as additional reference method to double-check lactose analysis in this product. Again, data of the HSQC method with reduced interscan delay are in good agreement with the results of the reference methods. As tested for product **6**, a prolonged interscan delay did not change the results.

**Figure 4:**
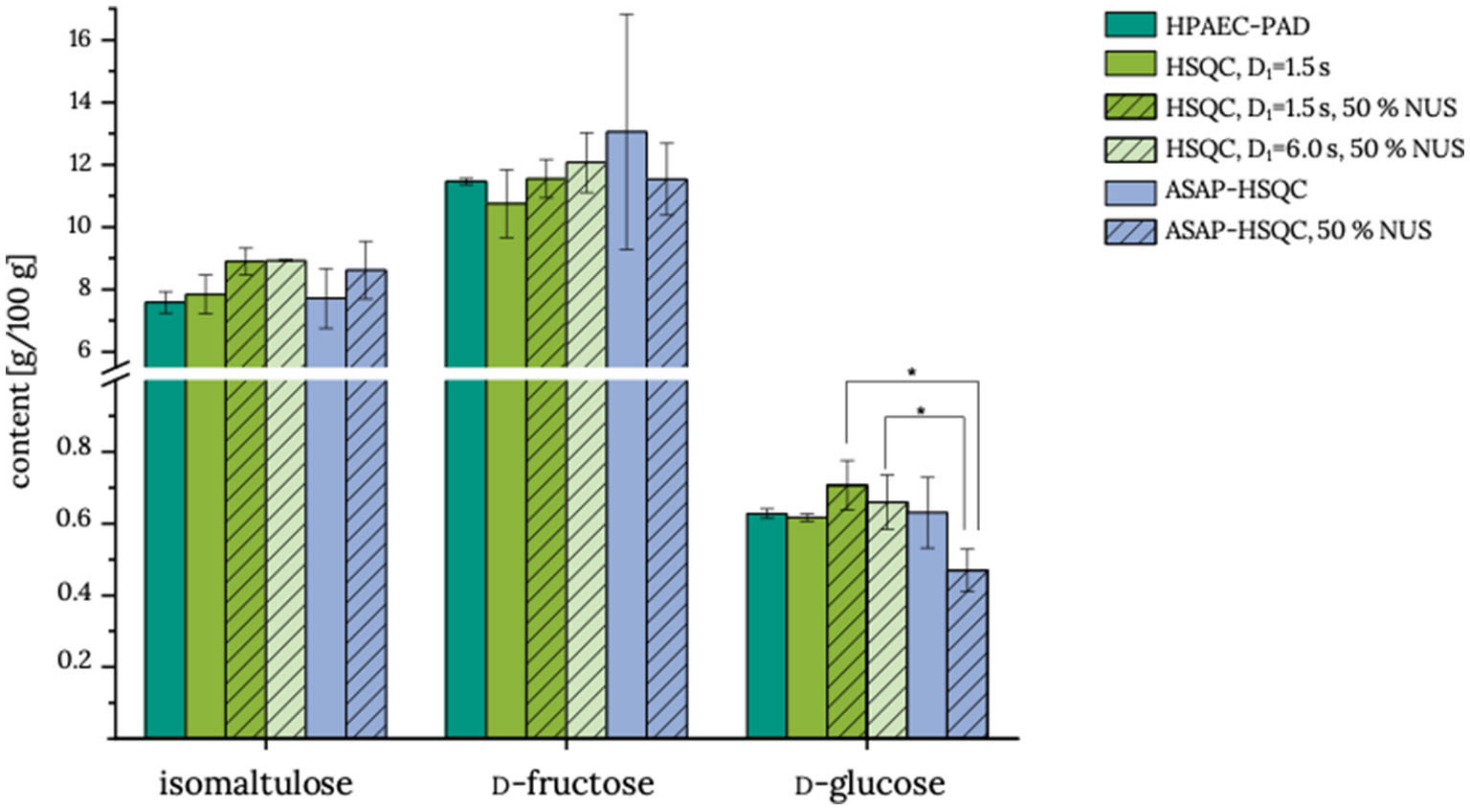
Contents of isomaltulose, d-fructose, and d-glucose in food product **3**. HPAEC-PAD, high-performance anion-exchange chromatography with pulsed amperometric detection; HSQC:, heteronuclear single quantum coherence; D_1_, interscan delay; NUS, non-uniform sampling; ASAP, acceleration by sharing adjacent polarization. All measurements were performed in triplicate determination, and the standard deviation is given. * indicates mean values that are statistically different (one-factor analysis of variance, α = 0.05, *post-hoc* Tukey test). Actual data are given in Supplementary Table S3.

Although reduction of the interscan delay already reduced the experiment time of a standard HSQC experiment to 53 min, application of NUS and ASAP-HSQC (with or without NUS) for the quantification of isomaltulose were evaluated in order to further reduce the experiment time. For this purpose, product **3** was analyzed in more detail (Figure 4). General applicability of ASAP-HSQC experiments for Carrez-clarified matrices has already been demonstrated in a previous study (15). Both the HSQC methods with NUS coupling and the ASAP-HSQC methods quantify isomaltulose, d-fructose, and d-glucose equivalently to the reference method HPAEC-PAD. However, it needs to be mentioned that analyses of d-fructose and d-glucose by using the ASAP-HSQC methods without NUS coupling suffer from high standard deviations compared to the other methods. Standard deviations for the analysis of isomaltulose by using ASAP-HSQC experiments appear to be higher as compared to the conventional HSQC experiment. The determined recoveries (98.5–105.3 %) of isomaltulose (Table 2) indicate sufficient accuracy of the results for all HSQC based methods. Thus, all HSQC based methods appear to be applicable to determine isomaltulose in food products; however, the results of the ASAP-HSQC methods are less precise than the results of the HSQC methods and, in case of glucose analysis, the determined levels appear to be somewhat low (ASAP-HSQC, NUS). Due to the high energy input during the measurement of the ASAP-HSQC methods, short-term overvoltages may occur, which may also cause the free induction decay to be erroneous. Thus, analysts need to decide whether analysis time or precision is the determining factor for their choice of method.

**Table 2:** Recoveries of isomaltulose determined by spiking food product 3.

**Method**	**Isomaltulose recovery [%] ± standard deviation [%]**
HPAEC-PAD	105.8 ± 1.6
HSQC, D_1_ = 1.5 s	105.3 ± 6.9
HSQC, D_1_ = 1.5 s, 50 % NUS	98.5 ± 2.7
HSQC, D_1_ = 6.0 s, 50 % NUS	100.8 ± 2.9
ASAP-HSQC	102.3 ± 24.8
ASAP-HSQC, 50 % NUS	105.6 ± 22.4

*HPAEC-PAD, high-performance anion-exchange chromatography with pulsed amperometric detection; HSQC, heteronuclear single quantum coherence; D_1_, interscan delay; NUS, non-uniform sampling; ASAP, acceleration by sharing adjacent polarization. All measurements were performed in triplicate determination, and the standard deviation is given*.

In conclusion, HSQC methods with shortened interscan delay were successfully applied to quantify isomaltulose in various food samples. Both buffer dissolved and Carrez clarified samples were considered. Application of NUS does not interfere with precision or accuracy, too, being a perfect addition to the experiment in order to further reduce analysis time. ASAP-HSQC methods are extremely fast (down to 6 min if coupled with NUS), but the results are less precise. Therefore, the ASAP-HSQC approach is probably more of a semi-quantitative approach and should not be used if critical levels need to be monitored. Overall, the HSQC experiment with reduced interscan delay and application of NUS appears to be the best choice to minimize analysis time but keeping high quality data in terms of precision. All time-accelerated HSQC methods tested here can quantify the selected analytes faster than the compared reference methods. However, HPAEC-PAD is certainly much more sensitive as compared to all HSQC approaches [for example, limits of detection: 0.01 μg/mL (HPAEC-PAD) vs. 0.12 mg/mL (HSQC, D_1_ = 1.5 s); limits of determination: 0.02 μg/mL (HPAEC-PAD) vs. 0.46 mg/mL (HSQC, D_1_ = 1.5 s)], generally limiting the applicability of the HSQC based methods to products that contain isomaltulose in %-quantities.

## Data Availability Statement

The raw data supporting the conclusions of this article will be made available by the authors, without undue reservation.

## Author Contributions

LF: conceptualization, methodology, investigation, laboratory work, and writing and editing. FR: methodology, investigation, and laboratory work. MB: conceptualization, methodology, writing and editing, and supervision. All authors contributed to the article and approved the submitted version.

## Funding

We acknowledge support by the KIT-Publication Fund of the Karlsruhe Institute of Technology.

## Conflict of Interest

The authors declare that the research was conducted in the absence of any commercial or financial relationships that could be construed as a potential conflict of interest.

## Publisher's Note

All claims expressed in this article are solely those of the authors and do not necessarily represent those of their affiliated organizations, or those of the publisher, the editors and the reviewers. Any product that may be evaluated in this article, or claim that may be made by its manufacturer, is not guaranteed or endorsed by the publisher.
