# Supplementary material for: Quantification of Isomaltulose in Food Products by Using Heteronuclear Single Quantum Coherence NMR-Experiments
Source: Front Nutr. 2022 Jun 27;9:928102. doi: 10.3389/fnut.2022.928102 (PMC9271938; doi:10.3389/fnut.2022.928102)
Supplement: Supplementary file 1 [file Data_Sheet_1.docx]

Quantification of isomaltulose in food products by using heteronuclear single quantum coherence NMR-experiments

L. Fels^1^, F. Ruf^1^, M. Bunzel^1*^

^1^ Department of Food Chemistry and Phytochemistry, Institute of Applied Biosciences, Karlsruhe Institute of Technology (KIT), Karlsruhe, Germany

*** Correspondence:**Corresponding Author
mirko.bunzel@kit.edu

# Supplementary

**Table S1: Longitudinal relaxation times (T_1_) of the analytes**

|  | T_1_ [s] |
| --- | --- |
| d-glucose | 2.1 |
| d-galactose | 1.6 |
| d-fructose | 1.8 |
| sucrose | 1.0 |
| lactose | 1.3 |
| maltose | 1.2 |
| isomaltulose | 0.9 |
| maltulose | 0.9 |

**Table S2: Contents of isomaltulose in g/100 g and (if applicable) d-fructose, d-glucose, and lactose (all g/100 g) in products 1-7 according to Figures 2 and 3**. GC-FID: gas chromatography with flame ionization detector; HPAEC-PAD: high performance anion exchange chromatography with pulsed amperometric detection, HSQC: heteronuclear single quantum coherence, D_1_: interscan delay. Analyses by using the reference methods were performed in duplicate using the half range to demonstrate the spread of the data. HSQC measurements were performed in triplicate determination, and the standard deviation is given.

|  |  | Enzymatic assay | GC-FID | HPAEC-PAD | HSQC,  D_1_=1.5 s | HSQC, D_1_=6.0 s (product 3)  D_1_=7.0 s (product 6) |
| --- | --- | --- | --- | --- | --- | --- |
| product 1 | isomaltulose | - | 94.73 ± 0.37 | 98.18 ± 0.26 | 100.46 ± 2.16 | - |
| product 2 | isomaltulose | - | 38.63 ± 0.03 | 39.27 ± 0.12 | 38.34 ± 0.52 | - |
| product 3 | isomaltulose |  | 9.25 ± 0.02 | 9.66 ± 0.01 | 9.46 ± 0.18 | 9.35 ± 0.29 |
|  | d-fructose | 12.32 ± 0.11 | 12.47 ± 0.07 | 12.70 ± 0.07 | 12.41 ± 0.33 | 12.38 ± 0.17 |
|  | d-glucose | 0.44 ± 0.00 | 0.43 ± 0.00 | 0.47 ± 0.01 | 0.47 ± 0.02 | - |
| product 4 | isomaltulose | - | 7.78 ± 0.09 | 8.20 ± 0.04 | 7.82 ± 0.31 | - |
| product 5 | isomaltulose | - | 8.09 ± 0.10 | 8.34 ± 0.16 | 7.74 ± 0.25 | - |
| product 6 | isomaltulose | - | 5.65 ± 0.00 | 5.62 ± 0.03 | 5.73 ± 0.05 | 5.74 ± 0.01 |
|  | lactose | 3.16 ± 0.02 | - | 3.37 ± 0.02 | 3.50 ± 0.03 | 3.47 ± 0.08 |
| product 7 | isomaltulose | - | 38.89 ± 0.17 | 39.40 ± 0.44 | 38.92 ± 0.54 | - |

**Table S3: Contents of isomaltulose, d-fructose, and d-glucose (g/100 g) in the food product 3 according to Figure 4.** HPAEC-PAD: high performance anion exchange chromatography with pulsed amperometric detection, HSQC: heteronuclear single quantum coherence, D_1_: interscan delay, NUS: non-uniform sampling, ASAP: acceleration by sharing adjacent polarization. All measurements were performed in triplicate determination, and the standard deviation is given.

|  | isomaltulose | d-fructose | d-glucose |
| --- | --- | --- | --- |
| HPAEC-PAD | 7.59 ± 0.34 | 11.46 ± 0.10 | 0.63 ± 0.01 |
| HSQC, D_1_=1.5 s | 7.84 ± 0.62 | 10.75 ± 1.09 | 0.62 ± 0.01 |
| HSQC, D_1_=1.5 s,  50 % NUS | 8.90 ± 0.42 | 11.55 ± 0.61 | 0.71 ± 0.07 |
| HSQC, D_1_=6.0 s,  50 % NUS | 8.93 ± 0.02 | 12.07 ± 0.96 | 0.66 ± 0.08 |
| ASAP-HSQC | 7.72 ± 0.96 | 13.06 ± 3.78 | 0.63 ± 0.10 |
| ASAP-HSQC,  50 % NUS | 8.62 ± 0.93 | 11.53 ± 1.15 | 0.47 ± 0.06 |
